# The Secretome Derived From Serum‐Free Media of SHED‐MSCs Can Modulate THP‐1‐Derived Macrophage Cells

**DOI:** 10.1111/jcmm.71063

**Published:** 2026-05-05

**Authors:** Azadeh Mohammad‐Hasani, Saeed Mohammadi, Mohsen Saeidi, Ali Fallah, Ayyoob Khosravi

**Affiliations:** ^1^ Stem Cell Research Centre, Biomedical Research Institute Golestan University of Medical Sciences Gorgan Iran; ^2^ Department of Molecular Medicine, Faculty of Advanced Medical Technologies Golestan University of Medical Sciences Gorgan Iran; ^3^ Natural and Medical Sciences Research Center University of Nizwa Nizwa Oman; ^4^ Department of Immunology, School of Medicine Golestan University of Medical Sciences Gorgan Iran; ^5^ Faculty of Biotechnology Amol University of Special Modern Technologies Amol Iran

**Keywords:** immunomodulation, macrophage polarisation, SFM‐derived secretome, SHED‐MSCs

## Abstract

Although culturing cells in serum‐free or low‐serum media may lead to reduced cell proliferation, it is crucial to minimise the presence of foreign proteins and other impurities that could compromise the validity of experimental findings on immunomodulation. The study aimed to investigate the immunomodulatory effects of the secretome derived from the serum‐free media (SFM) of stem cells obtained from human exfoliated deciduous teeth on THP‐1‐derived macrophage polarisation and the assessment of inflammatory and oxidative stress mediators. THP‐1 cells were polarised into M0 and M1 macrophages, and their differentiation was confirmed using flow cytometry to analyse specific markers of M0 (CD14 and CD68) and M1 (CD80 and CD86). The secretome was collected from SHED‐MSCs cultured for 48 h in serum‐free DMEM/F12 media. After treating THP‐1‐derived M0/M1 macrophages with SFM‐secretome from SHED‐MSCs, cells and supernatants were evaluated for immunosuppressive (anti‐inflammatory and antioxidant) and immunostimulatory (pro‐inflammatory and pro‐oxidant) indicators. Secretome treatment decreased the population of M1 macrophages, along with the expression of pro‐inflammatory and pro‐oxidative markers, including CD80, CD86, TNF‐α, IL‐12, NO, MDA and IL‐6R. The immunomodulatory effect of the secretome produced by SFM, which may help reduce inflammation, is demonstrated by an increase in M2 macrophages and anti‐inflammatory and antioxidant markers, including CD206, TGFβ‐2, IL‐10, TAC, CAT, SOD and ARG1. The secretome produced by SFM‐derived SHED‐MSCs may reduce the risk of introducing foreign proteins and other potentially hazardous chemicals and enhance the efficacy of cellular secretions for applications such as immunotherapy or regenerative medicine by modulating the immune system.

## 1 Introduction

Hundreds of millions of mesenchymal stem cells (MSCs) are needed for each cell therapy, which usually involves about 10 weeks of cell growth and expansion in a lab before implantation [1, 2, 3]. Stem cells migrate to the injury site in response to chemical signals after being introduced into the bloodstream [4, 5]. By repairing or replacing damaged cells, they then integrate into the injured area, differentiate into different cell types and support tissue regeneration [6, 7]. They also release bioactive compounds that influence local and systemic physiological processes [8]. Previous research has associated the therapeutic potential of MSCs with their ability to attach and differentiate into various cell types [9, 10, 11]. However, current studies suggest that the duration of MSC delivery is often too short to produce a significant effect [12, 13, 14, 15]. Moreover, the primary effects of MSCs are likely mediated through paracrine mechanisms [16], considering that less than 1% of MSCs survive beyond 1 week after systemic administration [17, 18].

A new form of biological regulation is emerging, where cells communicate by releasing various molecules. The molecules and chemicals released into the extracellular space during this process are collectively referred to as the secretome (SEC). These include nucleic acids, soluble proteins, lipids and extracellular vesicles, which can be classified as apoptotic bodies, microparticles or exosomes/EXOs [19, 20]. Odontogenic stem cells, derived from human exfoliated deciduous teeth (SHED), share many features with fibroblasts and can produce growth factors and cytokines essential for various biological functions [21, 22]. The secretome of SHED may influence macrophage polarisation.

To maintain balance, macrophages are essential for initiating inflammation‐related damage and then aiding in tissue repair. They exist in various forms, transitioning between the debris‐clearing and tissue‐remodelling M2 states and the proinflammatory M0 and M1 states. The shift from a pro‐inflammatory to an anti‐inflammatory environment is necessary for regeneration. Therefore, we used the human monocytic cell line THP‐1 in our study to investigate how human macrophages polarise. We focused on cell treatments that did not rely on live cells, leading our team to explore the paracrine components of SHED‐MSCs through three different approaches. Previously, we examined the effects of SHED‐MSCs (using a Transwell system) and their exosomes on monocyte‐derived macrophages [19, 23, 24]. In this study, we investigated the immunomodulatory capabilities of the secretome due to its advantages, such as ease of storage, high production and being a source of bioactive compounds, which are produced by SHED‐MSCs, on M0/M1 macrophages. To achieve this, M0/M1 macrophages derived from THP‐1 cells were treated with serum‐free media (SFM)‐secretome from SHED‐MSCs, and their effects on macrophage plasticity, surface molecule expression and cytokine release were assessed. We also measured gene expression and the levels of oxidative stress.

## 2 Materials and Methods

### 2.1 Preparation of SFM‐Derived Secretome

A 12‐year‐old girl at Golestan University of Medical Sciences provided the SHED‐MSCs for a previous study after obtaining written consent and following approved experimental procedures [25]. To transfer the SHED‐MSCs to a serum‐free environment, DMEM/F12 was used for culture, supplemented with 10% FBS and 1% PS; the FBS concentration was gradually decreased until it was eliminated. As the cells reached 50% confluency, this reduction was performed gradually, starting with 10% FBS, then decreasing to 5%, 2.5%, 1% and finally removing FBS completely. Each reduction was carried out every 48 h. After 48 h of growth in serum‐free medium, the supernatant was centrifuged for 10 min at 3000 rpm and identified as SFM‐secretome from SHED‐MSCs.

### 2.2 THP‐1 Monocyte Cell Line Differentiation to Pro‐Inflammatory Macrophages

THP‐1 cells were cultured in DMEM/F12 with 10% FBS and 1% PS at 37°C with 5% CO_2_. To minimise the effects of FBS during differentiation, the FBS concentration was gradually decreased from 10% to 5%, then to 2.5% and finally to 1%. Treating THP‐1 cells with 20 ng/mL of phorbol 12‐myristate 13‐acetate (PMA) and 0.05 mM of 2‐mercaptoethanol (2ME) led to the formation of differentiated M0 macrophages. Exposure to a complete medium containing 20 ng/mL of interferon‐gamma (IFNγ) and 100 ng/mL of lipopolysaccharide (LPS) induced the polarisation of M0 macrophages into M1 macrophages in an inflammatory state.

### 2.3 Treatment of Polarised THP‐1 Macrophage Cells

The research was conducted using six experimental groups. Two of these groups were regarded as negative controls, consisting of M0/M1 macrophages grown in complete media composed of DMEM/F12 with 1% FBS and 1% PS. The other two groups were treated with dexamethasone (DEX), which served as a positive control for the M0/M1 macrophages. The last two groups were test groups where complete media was used to treat M0/M1 macrophages with SFM‐secretome from SHED‐MSCs at a 3:1 ratio. The instructions in Table 1 were followed in carrying out the procedures for the six groups.

**TABLE 1:** Experimental setup for differentiating the THP‐1 monocyte cell line into M0/M1 macrophages and treating them with SFM‐secretome from SHED‐MSCs.

Number	Group	PMA + 2ME	LPS + IFN‐γ	DEX.	SFM‐secretome from SHED‐MSCs/total culture media
1	M0 Negative control (THP‐1 cells)	+	−	−	—
2	M1 Negative control (M0 cells)	−	+	−	—
3	M0 Positive control (M0 cells)	−	−	+	—
4	M1 Positive control (M1 cells)	−	−	+	—
5	M0 Test (M0 cells)	−	−	−	75%
6	M1 Test (M1 cells)	−	−	−	75%

### 2.4 Collecting the Supernatant and Harvesting the Cells

The supernatants from the M0 or M1 treatment wells were centrifuged for 10 min at 3000 rpm and then stored at −20°C. These supernatants were used to evaluate the levels of pro‐ and anti‐inflammatory cytokines, including transforming growth factor beta‐2 (TGFβ‐2), IL‐10, IL‐12 and tumour necrosis factor‐alpha (TNF‐α), as well as antioxidant markers such as superoxide dismutase (SOD) and catalase (CAT) activity, total antioxidant capacity (TAC), malondialdehyde (MDA) and nitric oxide (NO) levels. The M0/M1 macrophage cells that adhered to the bottom of the plate were washed twice with cold phosphate‐buffered saline (PBS: 0.1 M, pH 7.4). The cells were detached from the plates using cold ethylenediaminetetraacetic acid (EDTA: 10 mM) for 5 min. The isolated macrophage cells were centrifuged at 3000 rpm for 3 min and then preserved for future analysis of surface markers, such as the transmembrane proteins CD206, CD80 and CD86, using flow cytometry. Additionally, the expression of ARG‐1 and IL‐6R transcripts was assessed through real‐time PCR.

### 2.5 Pro‐Inflammatory and Anti‐Inflammatory Cytokines Measurements

A quantitative enzyme‐linked immunosorbent assay (ELISA) was used to quantify the levels of pro‐inflammatory cytokines, including TNF‐α and IL‐12β, as well as anti‐inflammatory cytokines, such as TGFβ‐2 and IL‐10. The ELISA assays were conducted using the ABclonal kit, and the results are presented as the amount of TNF‐α, IL‐12, TGFβ‐2 and IL‐10 in pg/mL. Each experimental condition was tested in triplicate.

### 2.6 Assessment of Oxidative Stress Levels

The production of NO in macrophages was measured by assessing nitrite levels in the culture supernatant after 48 h. MDA levels were evaluated based on their reaction with thiobarbituric acid (TBA) at temperatures between 90°C and 100°C, producing a pink pigment that has maximum absorption at 532 nm when MDA or similar compounds react with TBA. The results were expressed in nmol/mL of supernatant. An enzymatic antioxidant system acts as the initial defence against oxidative stress, helping to reduce the buildup of free radicals and protect cells from damage. Antioxidant enzymes such as SOD and CAT work to eliminate excess free radicals. SOD activity can be indirectly measured spectrophotometrically by monitoring the inhibition of pyrogallol autoxidation at 420 nm. One unit (U) of CAT activity is defined by the amount of hydrogen peroxide (H_2_O_2_) consumed per minute, specifically 1 μmol of H_2_O_2_ per minute. The TAC of the samples was assessed using the FRAP method, which measures the reducing power of divalent iron in a single electron transfer process. The levels of these markers were analysed using the KPG test kit from Karmania Pars Gene Co.

### 2.7 Cell Surface Flow Cytometry Staining

Analysis of cell surface markers was performed by using both conjugated and non‐conjugated antibodies with fluorescence techniques. To account for non‐specific fluorescence, suitable isotype controls were utilised. The cell surface analysis of THP‐1‐derived M0 macrophages involved a PE‐conjugated anti‐human CD14 antibody (catalogue no. #301806; Biolegend) and a FITC‐conjugated anti‐human CD68 antibody (Biolegend). For THP‐1‐derived M1 macrophages, a FITC‐conjugated anti‐human CD80 and a PE‐conjugated anti‐human CD86 (Biolegend) were used. Additionally, the CD80, CD86 and PE‐conjugated anti‐human CD206 (Biolegend) antibodies were used for surface analysis of M0/M1 macrophage cells treated with secretome. The effectiveness of M2 macrophage polarisation was evaluated by calculating the ratios of CD206/CD80 and CD86 for each differentiation. Samples were analysed using a BD Accuri flow cytometer (BD PharMingen, San Diego, CA) and BD Accuri C6 Flow analysis software.

### 2.8 The Expression Levels of *ARG1* and *IL‐6R* Transcripts

The impact of SFM‐secretome from SHED‐MSCs on the relative expression levels of ARG1 and IL‐6R was assessed using qRT‐PCR with the SYBR Green qPCR master mix YTA kit (catalogue no. YT2551) on an ABI 7500 qPCR machine. RNA was extracted using a FAVORGEN column kit (catalogue no. FABRK 000‐Mini), followed by cDNA synthesis with the Thermo Fisher Scientific kit (catalogue no. #K1621), according to the manufacturer's instructions. The experiment used specific primers for ARG1, IL‐6R and GAPDH. ARG1 (>NM_001244438.2) forward 5′‐GGTGGCAGAAGTCAAGAAGAAC‐3′ and reverse 5′‐GTGGTTGTCAGTGGAGTGTTG‐3′; IL‐6R (NM_001382774.1) forward 5′‐TCACTGTGTCATCCACGACG‐3′ and reverse 5′‐CTGGATTCTGTCCAAGGCGT‐3′; GAPDH (>NM_002046.7) forward 5′‐ACTTTGGTATCGTGGAAGGAC‐3′ and reverse 5′‐CAGTAGAGGCAGGGATGATG‐3′.

### 2.9 Statistical Analysis

Values were expressed as mean ± standard deviation (SD). A one‐way ANOVA was performed to assess the significance between different groups of parametric data. Analyses were conducted using GraphPad Prism software (version 8.4.2), and significance was set at *p* < 0.05. Each test was repeated three times.

## 3 Results

### 3.1 M0 and M1 Macrophage‐Specific Markers Confirmed by Flow Cytometry

The differentiation of THP‐1 monocytes into M0 macrophages was achieved by treating them with 20 ng of PMA for 24 h. Immunofluorescence staining showed increased levels of M0 macrophage markers CD14 and CD68. Subsequently, treating the M0 macrophages with 20 ng/mL of IFN‐γ and 100 ng/mL of LPS in DMEM/F12 medium for 24 h induced their transformation into M1 macrophages. Immunofluorescence analysis confirmed the expression of M1 macrophage‐specific markers CD80 and CD86.

### 3.2 The Secretome Reduced the Levels of Immunostimulatory Markers in THP‐1‐Derived Macrophage Cells

**FIGURE 1:**
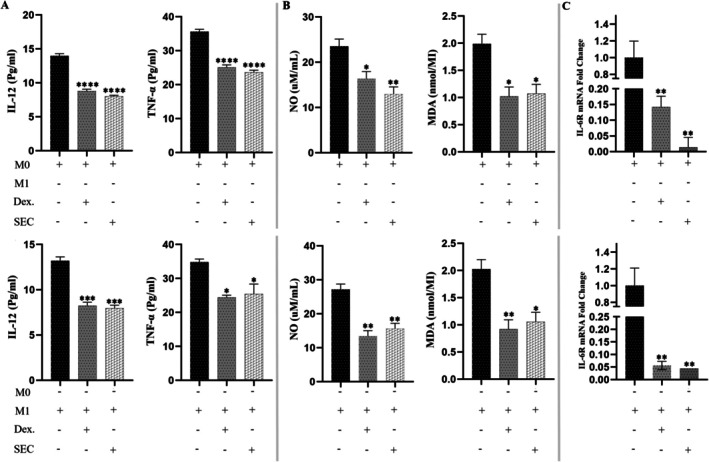
Effect of SFM‐secretome from SHED‐MSCs on immunostimulatory (pro‐inflammatory and pro‐oxidant) indexes in M0/M1 macrophage cells. (A) An ELISA assay was performed on M0/M1 macrophage cells treated with SFM‐secretome from SHED‐MSCs to assess the levels of the pro‐inflammatory cytokines TNF‐α and IL‐12. (B) The Griess assay and TBARS method were used to measure nitrite and malondialdehyde levels in both treated and untreated macrophage cells. (C) Treated macrophage cells showed a decrease in IL‐6R expression following SFM‐secretome from SHED‐MSCs treatment. All experiments were repeated in triplicate. Statistical significance was determined at *p* < 05. The mean ± SD of three independent experiments is shown for each bar. *p < 0.05; **p < 0.01; ***p < 0.001; ****p < 0.0001; ns: not significant. DEX, dexamethasone; SEC, secretome.

To assess the effects of SFM‐secretome from SHED‐MSCs on immunostimulatory markers in M0 and M1 macrophages, levels of IL‐12, TNF‐α, NO and MDA were measured in a culture medium treated for 48 h. The ELISA results showed that SFM‐secretome from SHED‐MSCs, similar to DEX, led to decreased levels of IL‐12 and TNF‐α (Figure 1A). Additionally, the Griess assay and TBARS methods indicated that the secretome reduced nitrite and MDA concentrations (Figure 1B). As shown in Figure 1C, macrophages treated with secretome and DEX exhibited lower IL‐6R expression levels compared to the control group (*p* < 0.05).

**FIGURE 2:**
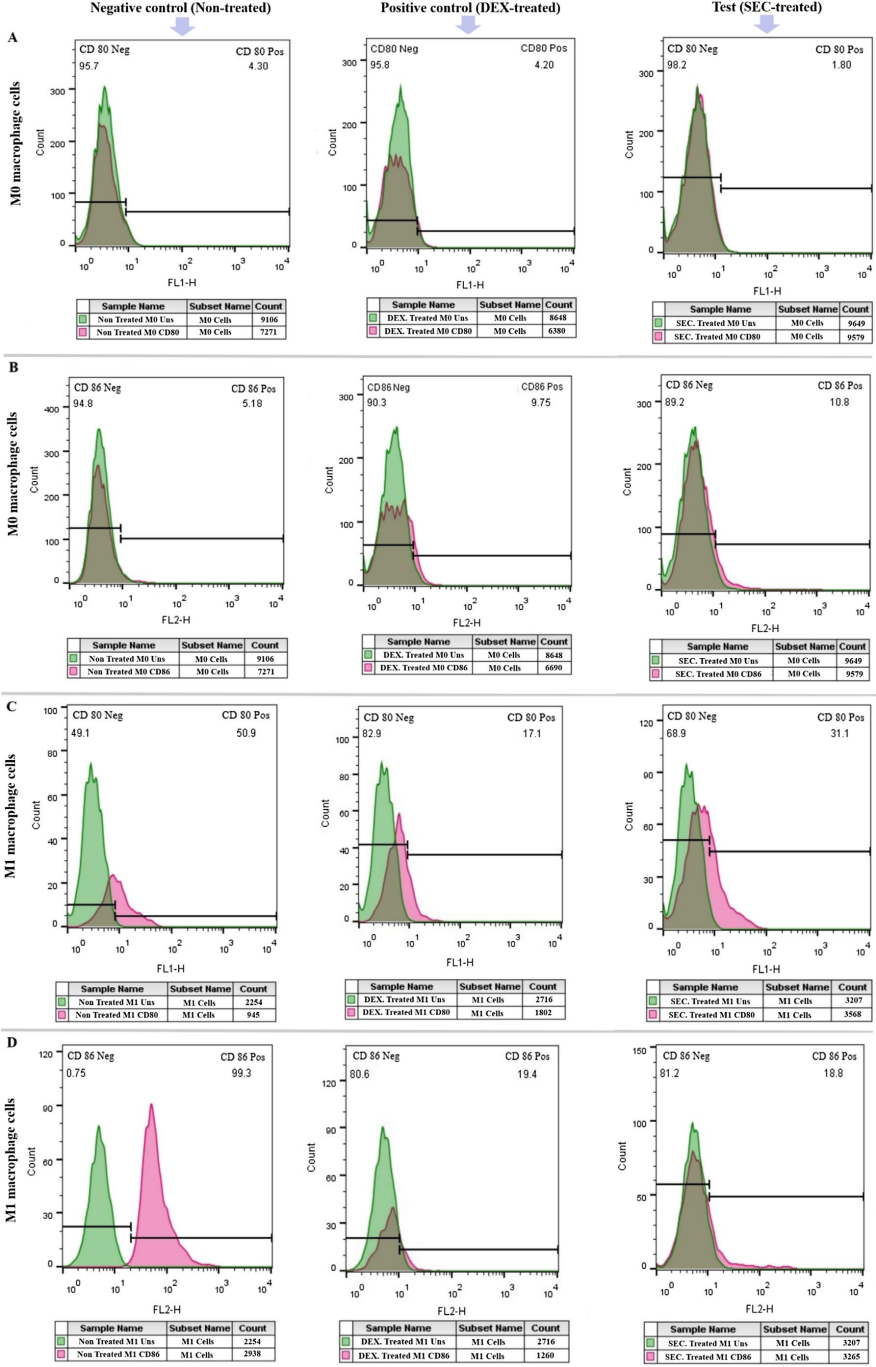
Effect of SFM‐secretome from SHED‐MSCs on the expression levels of M1‐specific markers (CD80 and CD86) as indicators of immunostimulation in M0/M1 macrophage cells. (A) The proportion of CD80‐positive cells decreases, while (B) CD86‐positive cells increase in M0 macrophages after exposure to SFM‐secretome from SHED‐MSCs, compared to the control group. (C, D) The proportion of cells expressing CD80 and CD86 in M1 macrophages decreases when exposed to the secretome, compared to the negative control. DEX, dexamethasone; SEC, secretome.

The expression levels of M1‐specific markers (CD80 and CD86) were also evaluated using flow cytometry and immunofluorescence analysis. The M0 group treated with SFM‐secretome from SHED‐MSCs showed reduced CD80 expression, similar to the DEX‐treated M0 group serving as a positive control (Figure 2A). Additionally, CD86 expression increased, like in the M0 positive control, as shown in Figure 2B. In the M1 group treated with SFM‐secretome from SHED‐MSCs, there was a significant decrease in both CD80 and CD86 expression, comparable to the M1 positive control group (Figure 2C,D).

### 3.3 SFM‐Secretome From SHED‐MSCs Increased Immunosuppressive Indexes in M0/M1 Polarised Macrophage Cells

**FIGURE 3:**
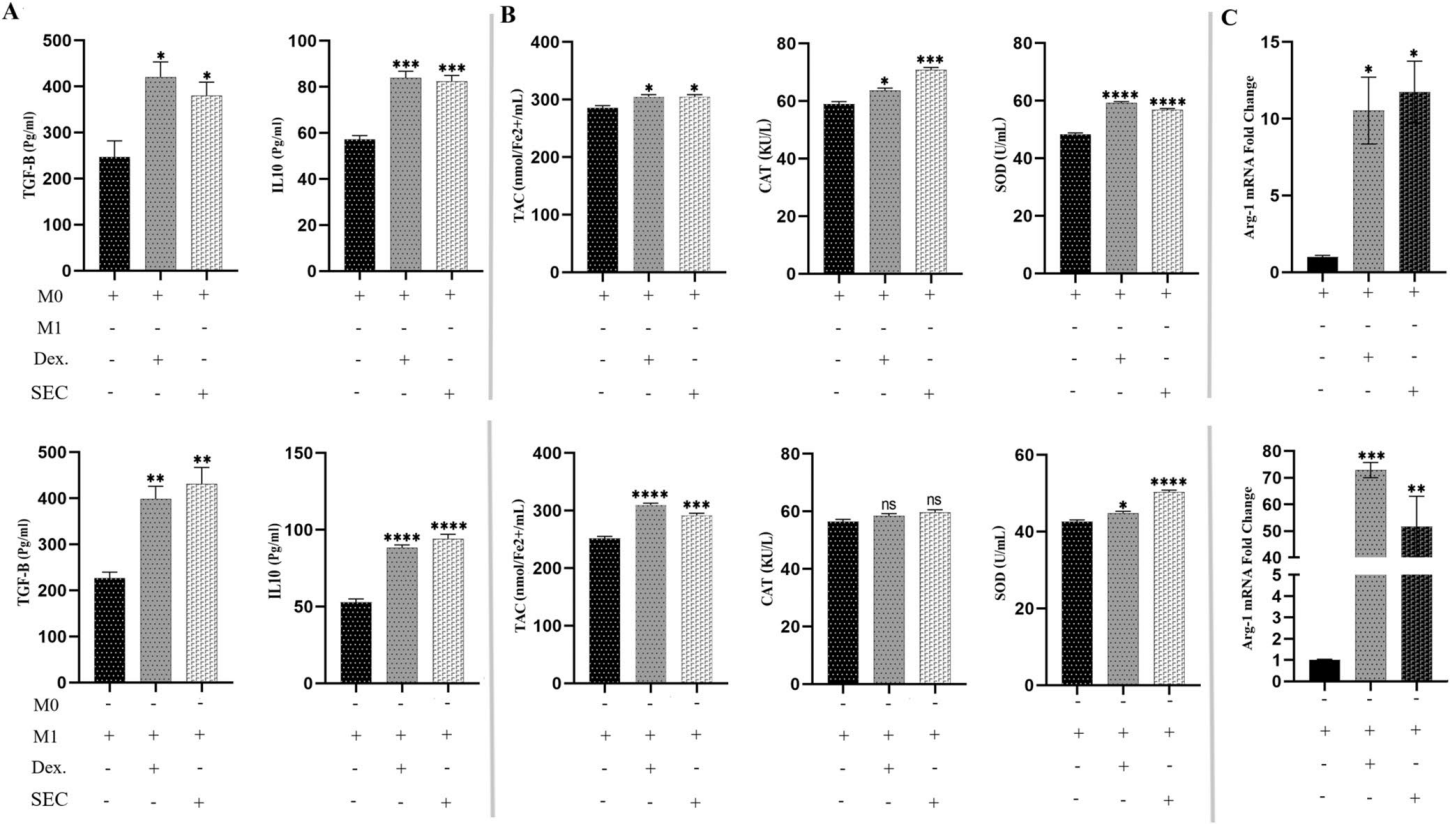
Effect of SFM‐secretome from SHED‐MSCs on immunosuppressive (anti‐inflammatory and antioxidant) markers in M0/M1 macrophage cells. (A) The levels of TGFβ‐2 and IL‐10, anti‐inflammatory cytokines, were measured in M0/M1 macrophages before and after treatment using an ELISA test. (B) The FRAP method, CAT activity assay and SOD activity assay were conducted on M0/M1 macrophage cells treated with SFM‐secretome from SHED‐MSCs, along with control groups. The purpose was to evaluate their ability to reduce the ferric‐tripyridyltriazine (Fe^3+^‐TPTZ) complex to ferrous‐TPTZ (Fe^2+^‐TPTZ) through the antioxidants in the sample at low pH, as well as their capacity to decompose H_2_O_2_ and pyrogallol. (C) Treatment with SFM‐secretome from SHED‐MSCs resulted in increased ARG1 expression in M0/M1 macrophage cells. The experiment was performed in triplicate, and statistical significance was assessed with a *p* value of less than 0.05. Each bar in the graph shows the mean ± standard deviation of three independent experiments. Significance levels are indicated as follows: *p < 0.05; **p < 0.01; ***p < 0.001; ****p < 0.0001; ns: non‐significant. DEX, dexamethasone; SEC, secretome.

To evaluate the impact of SFM‐secretome from SHED‐MSCs on immunosuppressive markers in M0 and M1 macrophages, the levels of TGFβ‐2, IL‐10, TAC, CAT, SOD and ARG1 in culture medium treated with the secretome for 48 h were measured. ELISA results showed that SFM‐secretome from SHED‐MSCs treatment, similar to DEX, increased levels of TGFβ‐2 and IL‐10 (Figure 3A). Additionally, the Griess test and TBARS method indicated a nitrite reduction and MDA levels following SFM‐secretome from SHED‐MSCs treatment (Figure 3B). The study also examined ARG1 expression, a key anti‐inflammatory marker associated with M2 macrophages. As shown in Figure 3C, the findings demonstrated that macrophages treated with secretome and DEX exhibited higher ARG1 levels compared to the control group (*p* < 0.05).

**FIGURE 4:**
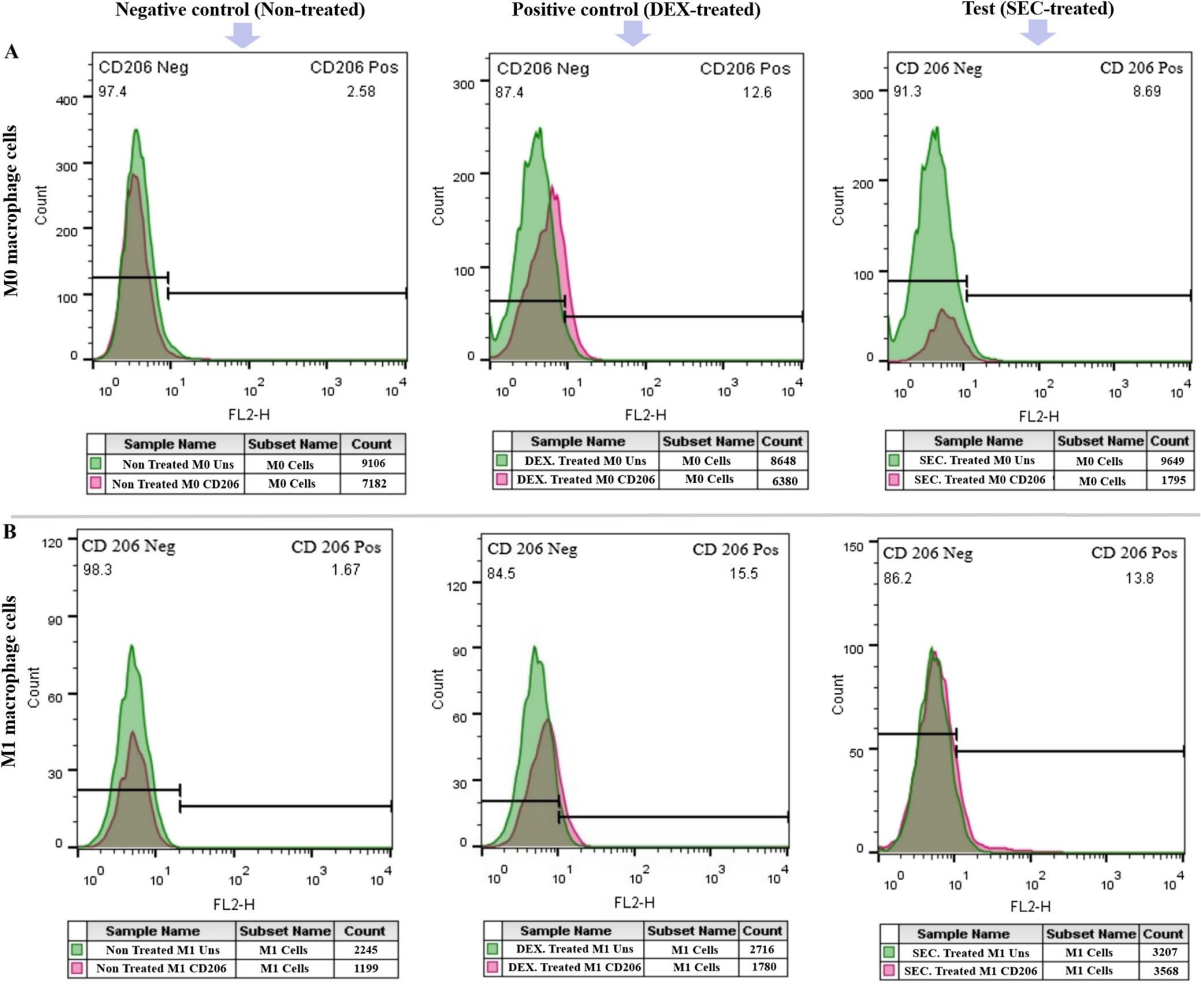
Impact of SFM‐secretome from SHED‐MSCs on the expression levels of the M2‐specific marker (CD206) as an indicator of immunosuppression. The percentage of CD206‐positive cells in (A) M0 and (B) M1 macrophage cells rose after treatment with the secretome when compared to the control group. DEX, dexamethasone; SEC, secretome.

To further explore immunosuppressive markers, immunofluorescence staining was performed to assess the expression of CD206, a marker specific to M2 macrophages. Treatment with SFM‐secretome from SHED‐MSCs resulted in an increase in CD206 expression in both M0 (Figure 4A) and M1 (Figure 4B) macrophages, indicating a shift toward the M2 macrophage phenotype.

## 4 Discussion

The immunoregulatory functions of cells, their secretome and exosomes are important in therapeutic uses, especially in regenerative medicine and immune modulation [26]. Concerning the significance of non‐cell‐based therapies, our team has studied the paracrine factors of SHED‐MSCs through three concurrent projects. The first project examined how SHED‐MSCs interact with macrophage cells derived from THP‐1 using a transwell system [24]. Later, to improve our understanding of the natural immunomodulatory features of SHED‐MSCs, their exosomes were isolated and characterised, and the impact of these nanovesicles on THP‐1‐derived macrophage cells was evaluated [19, 23].

**FIGURE 5:**
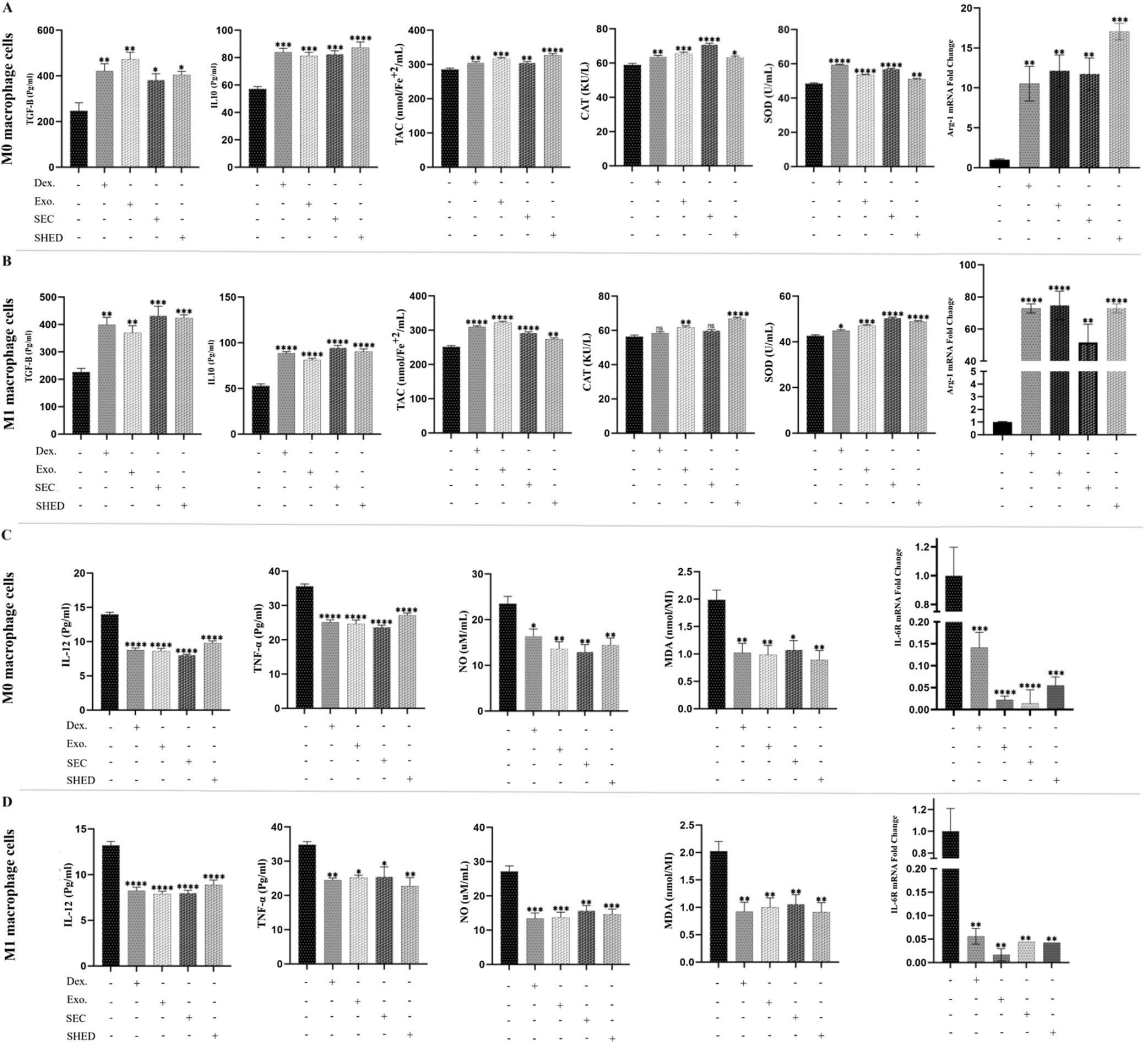
Comparison of how various components derived from SHED‐MSCs modulate immune responses in both resting (M0) and activated (M1) macrophage states. (A) Shows the Immunosuppressive Indexes for untreated and treated M0 macrophages, demonstrating that exosomes, secretome and SHED‐MSCs (using the transwell system) enhance the anti‐inflammatory and antioxidant properties of these cells. (B) Focuses on the Immunosuppressive Indexes in untreated and treated M1 macrophages, showing that similar treatments lead to increased anti‐inflammatory markers and antioxidant indicators. (C) Illustrates the Immunostimulatory indices for untreated and treated M0 macrophages, indicating that these treatments reduce pro‐inflammatory and pro‐oxidant activities in M0 macrophages by lowering the levels of corresponding markers. Finally, (D) presents the Immunostimulatory indices for untreated and treated M1 macrophages, highlighting the effects of exosomes, secretome and SHED‐MSCs on pro‐inflammatory and pro‐oxidant indices. The results show a decrease in these indices following treatment. The experiment was performed in triplicate, and statistical significance was assessed with a *p* value of less than 0.05. Each bar in the graph shows the mean ± standard deviation of three independent experiments. Significance levels are indicated as follows: *p < 0.05; **p < 0.01; ***p < 0.001; ****p < 0.0001; ns: non‐significant. DEX, dexamethasone; EXO, exosome; SEC, secretome; SHED, stem cells from human exfoliated deciduous teeth.

In this project, we explored the immunomodulatory effects of the secretome derived from SHED‐MSCs on M0/M1 macrophages, considering the benefits of the secretome, including its storage advantages, potential for large‐scale production and role as a source of bioactive factors. An experimental model of THP‐1‐derived M0/M1 macrophage cells provides evidence supporting our findings that treatment with SFM‐secretome from SHED‐MSCs caused a significant reduction in the levels of immunostimulatory markers, including IL‐12 and TNF‐α (Figure 1A); NO and MDA (Figure 1B); CD80 (Figure 2A,C); and CD86 (Figure 2D); as well as the mRNA expression of IL‐6R (Figure 1C). An increase in CD86 expression was observed in M0 macrophages treated with secretome, which could steer these cells toward an alternative anti‐inflammatory state rather than the typical M2 phenotype. Additionally, Mohammad‐Hasani et al. noted this dual activation pattern in the active secretome using transwell co‐culture [24], reflecting adaptive regulation influenced by macrophage signals that promote STAT3/6 pathways for M2 skewing while partially engaging NF‐κB/IRF5 for temporary M1 modulation [27, 28]. The versatility of SFM‐secretome from SHED‐MSCs is demonstrated by its ability to activate different M2 subtypes (M2a, M2b, M2c) through the release of cellular products such as soluble factors and extracellular vesicles. Conversely, there was an increase in the expression of immunosuppressive markers, including IL‐10 and TGFβ‐2 (Figure 3A); TAC, CAT and SOD (Figure 3B); CD206 (Figure 4); and ARG1 mRNA (Figure 3C), which induce an anti‐inflammatory response similar to that observed with amniotic fluid MSCs‐CM in a mouse model of colitis [29]. Overall, our research shows that secretome, like isolated exosomes and mother cells, can independently influence M0 and M1 macrophage polarisation while reducing their inflammatory (Figure 5A,B) and oxidative (Figure 5C,D) features.

The immunomodulatory effects on macrophage phenotype are driven by a wide range of secretome components that coordinate complex paracrine signalling. Anti‐inflammatory cytokines such as IL‐10, TGF‐β2, IL‐4, PGE2, HGF and PEDF, detected via ELISA in our study, encourage the activation of M2 subtypes (M2a, M2b, M2c) by increasing ARG1 mRNA expression (Figure 3C) and decreasing IL‐6R through negative regulation of Notch1/NF‐κB and JAK/STAT pathways, thus reducing macrophage sensitivity to inflammatory signals [27, 28, 30, 31]. These soluble factors correspond with our observed increases in IL‐10/TGF‐β2 (Figure 3A) and decreases in TNF‐α/IL‐12 (Figure 1A), supporting AKT‐mediated repair phenotypes. Chemokines like CCL2, which form heterodimers with CXCL12 to activate CCR2 for M2 polarisation, and CX3CL1, which targets CX3CR1 to influence microglial activation and phagocytosis, facilitate complex chemotactic interactions beyond direct signalling [32, 33, 34]. Extracellular vesicles (EVs) further enhance this process by transferring functional mitochondria and activated STAT3, boosting ARG1 induction and promoting anti‐inflammatory states via CD9/CD81 interactions with FcγR/TLR4 to inhibit LPS activation [34, 35, 36, 37]. Collectively, these components combine NF‐κB/IRF5 inhibition (partially modulating M1) with STAT3/6 promotion (M2 dominance), as seen by increased CD206 (Figure 4) and decreased CD80/CD86 expression (Figure 2A,C,D), positioning SHED‐MSC secretome as a versatile acellular immunomodulator [19, 37, 38, 39, 40].

Oxidative stress regulation in macrophages is mainly controlled by secretome components that restore redox balance and reduce ROS/RNS‐driven inflammation. Antioxidant enzymes like SOD and CAT, along with low‐ROS metabolites from glycolysis, increase TAC while decreasing MDA and NO levels in M1 macrophages (Figure 1B). Soluble cytokines like TGF‐β2 and IL‐10 activate the Nrf2/ARE pathway to boost cellular defences and suppress iNOS/NOX1‐5 enzymes responsible for excess ROS/RNS [15, 41, 42]. As shown in Figure 5, our results indicate significant CAT increases in exosome‐treated macrophages and SHED‐MSC‐treated macrophages in the transwell system, emphasising its context‐dependent ability to neutralise lipid peroxidation and nitrosylation [19, 42, 43]. PGE2 and HGF further inhibit pro‐oxidant NF‐κB/STAT1 activation in M1 states, while promoting STAT3/6/PPARγ in M2 states to reduce apoptosis via cytochrome C/caspases 3/7 and ASK1/JNK pathways, helping maintain bactericidal function while avoiding secondary tissue damage [38, 44, 45]. EVs assist by delivering miRNAs and mitochondria to suppress mitochondrial (MOS) and LPS‐induced (LOS) ROS/NO through AMPK/SIRT1/Hippo‐YAP regulation, consistent with our multi‐readout findings of reduced TNF‐α/IL‐6/IL‐1β levels and increased IL‐10/TGF‐β/ARG1 levels (Figure 1A,C) [19, 36, 46]. This integrated regulation, evidenced by decreased pro‐oxidant markers and increased antioxidant indicators, supports reparative phenotypes (IL‐10/TGF‐β/ARG1/CAT/SOD/TAC) vital for tissue remodelling and angiogenesis, highlighting the therapeutic potential of SFM‐derived secretome in oxidative‐related diseases [45, 47].

Beyond canonical proteins and EVs, non‐protein secretome components, such as low‐molecular‐weight compounds, diffusible factors, lipids and metabolic byproducts, play crucial roles in macrophage reprogramming through biophysical and metabolic modulation. Low‐molecular‐weight metabolites (e.g., lactate, short‐chain fatty acids < 1 kDa) act as direct ROS scavengers and alternative energy sources, shifting M1 glycolysis to M2 fatty acid oxidation (FAO) to reduce pro‐inflammatory responses, as shown by our decreased MDA/NO levels (Figure 1B) [19, 41]. Diffusible elements, including uncharged metabolites and gases from heme oxygenase (e.g., CO), facilitate rapid paracrine signalling across membranes, quickly activating Nrf2 to enhance TAC/SOD/CAT in treated macrophages (Figure 5). Lipids such as sphingolipids and phospholipids modify membrane fluidity and receptor activity, upregulating PPARγ/STAT6 to promote M2 polarisation while inhibiting lipid peroxidation pathways, consistent with increased ARG1/CD206 (Figures 1C and 5) and decreased CD80/CD86 levels (Figure 2A,C,D). Metabolic products from SHED‐MSC respiration (e.g., succinate, α‐ketoglutarate) and EV‐mediated mitochondrial transfer help regulate redox balance, suppressing NOX/iNOS‐driven MOS/LOS formation to prevent cytochrome C‐mediated apoptosis and support tissue repair [48]. Notably, the active secretome's distinct CAT enhancement (Figure 5B) illustrates adaptive metabolic synergy, reducing SFM contamination risks for safer acellular therapies. Future metabolomic profiling will clarify their quantitative effects.

In this research, FBS was gradually removed to eliminate the interference of bovine cellular contents. Serum‐free or low‐serum conditions may reduce overall cell proliferation. However, this approach minimises the risk of extraneous proteins and other contaminants that could potentially skew experimental results regarding immune modulation, thereby enhancing the functional applicability of the secretome for targeted applications such as immunotherapy and regenerative medicine. Lee et al. illustrated that adipose‐derived mesenchymal stem cells (ADSCs) cultured in an SFM exhibited a more consistent population doubling time following passaging and generated more cells in a shorter timeframe compared to those maintained in FBS. Additionally, ADSCs cultured in SFM demonstrated reduced levels of cellular senescence, diminished immunogenicity and enhanced genetic stability relative to their FBS‐cultured counterparts. Conversely, the analysis of mRNA and protein expression in ADSCs grown in FBS revealed that genes associated with apoptosis, immune response and inflammation were significantly more active [49].

The immunoregulatory functions of cells, secretomes and exosomes offer a unique advantage [50]. Cells display dynamic responses, enabling direct interactions and paracrine effects, and have differentiation potential that can be harnessed for therapy [51]. The secretome, which includes a wide range of components, provides a broad spectrum of effects and may offer a more comprehensive approach; however, it lacks the targeting accuracy of exosome therapy [52]. Exosomes allow for precise delivery of specific cargo, demonstrate greater stability than whole‐cell therapies and pose a lower risk of immunological rejection because they are acellular [53]. Regarding stability and safety, cells have a more complex safety profile since they pose risks like uncontrolled growth or differentiation, which require careful monitoring [54]. The stability of the secretome depends on the methods used for isolating its components; while it is generally safer than whole‐cell therapies, it may need careful formulation to reduce adverse reactions [55]. Exosomes are known for their high stability and lower risk of tumour formation or ectopic tissue growth compared to living cell therapies [56]. Regulatory challenges differ among these approaches: exosomes and secretomes usually face a more straightforward regulatory path than live cell therapies, due to their acellular nature, though issues relating to standardisation and characterisation remain [57]. In contrast, cells undergo strict regulatory oversight because of their complexity and potential risks associated with their use [58]. In summary, each component offers unique advantages in immunoregulation, with the choice of approach depending on specific clinical settings, desired outcomes, safety factors and regulatory considerations [59]. Notably, our SFM‐derived SHED‐MSC secretome promotes M2 polarisation (increased CD206/ARG1), while reducing M1 markers (decreased CD80/CD86), diminishing pro‐inflammatory cytokines (TNF‐α, IL‐12) and decreasing oxidative stress (NO, MDA), thereby lowering contamination risks for improved immunotherapy and regenerative applications.

While our findings demonstrate promising immunomodulatory effects of SHED‐MSC secretome in vitro, the study is limited by its design using THP‐1‐derived macrophages. Future in vivo studies in animal models are essential to validate clinical relevance, therapeutic efficacy and potential off‐target effects.

## 5 Conclusion

Mesenchymal stem cells can directly interact with immune cells through surface receptors and/or secrete various factors that influence the activity of nearby immune cells via paracrine signalling. Certain therapeutic cells have the potential to differentiate into different cell types crucial for tissue repair while also affecting local immune responses. Additionally, living cells have a dynamic ability to respond to their environment by altering their secretion profiles in response to feedback from surrounding tissues.

The secretome contains numerous factors that can have synergistic or antagonistic effects on the immune system. Within the secretome, cytokines may either promote or inhibit inflammatory responses, while growth factors and chemokines assist in tissue repair and also influence the recruitment and activation of immune cells. Because of its complex nature, the secretome can produce broad immunomodulatory effects, surpassing those of individual components like exosomes. Exosomes play a key role in modulating immune responses through antigen presentation and regulation of T cells, B cells, dendritic cells and macrophages. Exosomes derived from MSCs often exhibit anti‐inflammatory effects by delivering microRNAs and proteins that suppress pro‐inflammatory cytokines, thereby aiding tissue repair and supporting immune responses that promote healing.

In summary, treatment with the SFM‐derived secretome of SHED‐MSCs substantially reduced the M1 macrophage population (lowered CD80, CD86, TNF‐α, IL‐12, NO, MDA and IL‐6R mRNA) while increasing the M2 population (higher CD206, ARG1 mRNA, TGF‐β2, IL‐10, TAC, CAT and SOD) in THP‐1‐derived models, promoting an anti‐inflammatory and antioxidant shift. These effects, mediated by key components such as cytokines (IL‐10, TGF‐β2), EVs/miRNAs (e.g., hsa‐miR‐1260a inhibiting NF‐κB/STAT1), low‐molecular‐weight metabolites (e.g., lactate for ROS scavenging), lipids (sphingolipids modulating inflammation) and diffusible factors (PGE2/TSG‐6 activating STAT3/6 and Nrf2 pathways), highlight the secretome's adaptive paracrine role in macrophage reprogramming [23, 24]. Our study offers new insights by evaluating the complete SFM‐derived secretome, not just exosomes, in THP‐1 models, reducing contamination for clinical use. Unlike exosome‐focused research [19, 57], we show simultaneous modulation of inflammation and oxidative stress, with active versus passive secretome displaying adaptive paracrine crosstalk (e.g., CCL2/CXCR2 for M2 recruitment). This comprehensive SFM approach addresses gaps in earlier FBS‐dependent studies, boosting therapeutic potential [5, 43]. These findings provide valuable insights for developing innovative treatments that target specific disease pathways involving macrophage biology. The secretome from SHED‐MSCs offers a promising method for polarising macrophages and modulating immune responses for therapy. However, while promising in vitro, further in vivo research is essential to confirm safety and efficacy. Continued investigation is needed to explore their full potential and improve their application in clinical settings.

## Author Contributions

**Azadeh Mohammad‐Hasani:** validation (equal), visualization (equal), writing – original draft (lead), writing – review and editing (equal). **Saeed Mohammadi:** data curation (equal), software (equal), writing – review and editing (equal). **Mohsen Saeidi:** investigation (equal), supervision (equal), writing – review and editing (equal). **Ali Fallah:** formal analysis (equal), software (equal), writing – review and editing (equal). **Ayyoob Khosravi:** methodology (equal), project administration (equal), writing – review and editing (equal).

## Funding

This study was supported by a grant from the Golestan University of Medical Sciences (grant number: 112570).

## Ethics Statement

The Ethics Committee approved this study at the Golestan University of Medical Sciences (Code# IR.GOUMS.REC.1401.394).

## Conflicts of Interest

The authors declare no conflicts of interest.

## Data Availability

All data generated or analysed during this study are included in this published article.
